# SpaceWalker enables interactive gradient exploration for spatial transcriptomics data

**DOI:** 10.1016/j.crmeth.2023.100645

**Published:** 2023-11-15

**Authors:** Chang Li, Julian Thijssen, Thomas Kroes, Mitchell de Boer, Tamim Abdelaal, Thomas Höllt, Boudewijn Lelieveldt

**Affiliations:** 1 Department of Radiology, Leiden University Medical Center, 2333 ZA Leiden, the Netherlands; 2 Delft Bioinformatics Lab, INSY, TU Delft, 2628 XE Delft, the Netherlands; 3 Computer Graphics and Visualization, INSY, TU Delft, 2628 XE Delft, the Netherlands

**Keywords:** data visualization, visual analytics, spatial transcriptomics, gene expression gradients

## Abstract

In spatial transcriptomics (ST) data, biologically relevant features such as tissue compartments or cell-state transitions are reflected by gene expression gradients. Here, we present SpaceWalker, a visual analytics tool for exploring the local gradient structure of 2D and 3D ST data. The user can be guided by the local intrinsic dimensionality of the high-dimensional data to define seed locations, from which a flood-fill algorithm identifies transcriptomically similar cells on the fly, based on the high-dimensional data topology. In several use cases, we demonstrate that the spatial projection of these flooded cells highlights tissue architectural features and that interactive retrieval of gene expression gradients in the spatial and transcriptomic domains confirms known biology. We also show that SpaceWalker generalizes to several different ST protocols and scales well to large, multi-slice, 3D whole-brain ST data while maintaining real-time interaction performance.

## Highlights

• A visualization tool for exploring spatial transcriptomics data in 2D and 3D
• On-the-fly retrieval of tissue structure and localized gene expression gradients
• Scales to large, multi-slice, 3D whole-brain data with real-time performance
• Generalizes across spatial transcriptomics protocols

## Motivation

Spatial transcriptomics (ST) enables profiling of the expression of hundreds of genes in tissue sections down to the level of single cells in their tissue environment. The gradient structure of ST data is particularly interesting for tissue biology because spatial gene expression gradients often represent tissue compartment edges, whereas in the single-cell transcriptomic domain, gene expression gradients may represent cell-type differences and smooth phenotypic transitions. Various computational approaches have been developed to extract information from either the spatial domain or the gene expression domain individually. However, integrative biological interpretation of expression gradients in single-cell and ST data spaces remains challenging. Many prior ST analysis pipelines are script based, lack interactive exploration facilities, and do not have specific facilities for automatic identification of localized expression gradients.

## Graphical abstract

**Figure:**
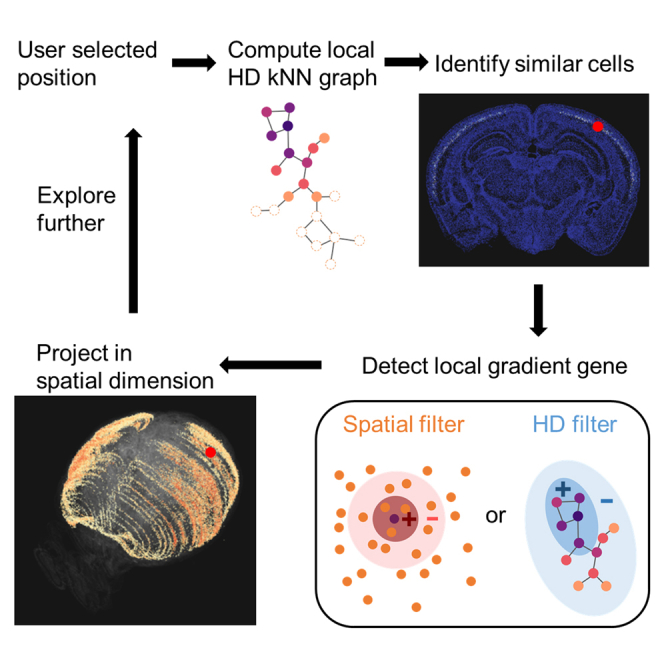

## Teaser

Li et al. describe SpaceWalker, a visual analytics tool for exploring the tissue architecture and local gradient structure of spatial transcriptomics data in 2D and 3D. Their tool enables the user to gain insight into the spatial patterning and the associated gene expression gradients in the tissue.

## Introduction

Spatial transcriptomics (ST) enables profiling the expression of hundreds of genes at the level of single cells in tissue sections, thus providing new opportunities to understand tissue biology by combining single-cell gene expression profiles and their corresponding spatial context in the tissue.^1^ For single cells, these high-dimensional (HD) gene expression profiles enable detailed characterization of cell types, cell states, and cell maturation.^2^ Data visualization methods, and specifically dimensionality reduction (DR) techniques, are extensively used to help understand complex HD data by giving a meaningful low-dimensional (LD) representation of these HD spaces. This typically consists of generating and visualizing an LD map in which distances, similarities, or neighborhood relations from the HD data space are preserved in the LD space.^3^ DR embeddings give insight into the structure of the data, showing clusters and generic trends in the data. Besides analysis of which cell types are present in a sample, trajectory inference approaches aim to computationally derive transitions between cell types and states and order cells along a trajectory topology. This allows investigating cellular dynamics, where expression gradients in one or more genes encode biologically relevant state transitions and cell variability.^4^ DR techniques are typically applied to reduce data complexity, and algorithms like k-nearest neighbor (kNN) graph construction and clustering techniques are used to extract the topological structure of the data.^2^

As single-cell RNA sequencing (scRNA-seq) does not support characterizing the spatial organization and interactions between cells, ST adds a spatial dimension to single-cell analysis, enabling the study of cell-cell interactions, tissue architecture, and coupled cell development and migration trajectories in the tissue.^5^ Understanding the structure of the spatial map or non-linear DR embeddings often requires localized methods to visualize the features driving the map structure at that particular location.^6^ Coloring the cells in these 2D maps with gene expression values is commonly used in single-cell analysis to visualize the underlying expression patterns, but genes of interest have to be manually selected, and it is not possible to reveal how cells are connected in HD space.

Various computational approaches have been developed to extract information from either the spatial domain or the gene expression domain. For instance, hidden Markov random fields were used to integrate scRNA-seq data and spatial neighborhood information.^7^^,^^8^^,^^9^ In addition, several methods exist for spatial gene expression analysis. SpatialDE^10^ applied a Gaussian process regression to identify genes with correlated expression levels in the spatial domain. Trendsceek^11^ detected spatial dependency of gene expressions using a marked point process. Although these approaches enable the identification of spatial gene expression variation, localized gene expression analysis methods are lacking. Furthermore, these computational strategies are script based and lack interactive data exploration facilities with a direct feedback loop to the user.

Explorative analysis and visualization of ST data allow for generating hypotheses on tissue biology. Integrative toolboxes such as Giotto^12^ and Squidpy^13^ allow researchers to interactively visualize and analyze spatial data. Cytosplore^14^ is software that uses t-distributed stochastic neighbor embedding (t-SNE)^15^ projection as the main view for real-time interactive visual exploration in the single-cell analysis domain. In addition to efficiently processing large-scale mass cytometry data, it also provides clustering techniques and supports multiple linked views between the map structure and the feature space. Based on Cytosplore, Cytosplore Transcriptomics^16^ provides interactive analysis for scRNA-seq data. CELLxGENE^17^ is a web-based interface aimed at interactive exploration of HD single-cell datasets. It enables collaborative analysis between experimentalists and bioinformaticians. Despite the active development of interactive visualization tools, interactive biological interpretation of single-cell HD and spatial data remains challenging. The relationship between HD single-cell data and 2D maps has not been fully explored.

In this work, we present SpaceWalker, a visual analytics tool for exploring the gradient structure of ST data. Specifically, we focus on interactive exploration of localized gene expression gradients: these are particularly interesting for tissue biology, as spatial gene expression gradients often represent tissue compartment edges, whereas in the HD single-cell transcriptomic domain, they represent cell-type differences and smooth phenotypic transitions between them. In SpaceWalker, the user can be guided by the local intrinsic dimensionality of the HD data to interactively pick seed locations for a series of neighborhood searches on the kNN graph. The results of these searches, i.e., localized HD neighborhood “flood fills,” are then projected onto the 2D spatial map in real time, revealing the spatial topology of the HD kNN graph. These localized HD topology approximations then serve as input for gradient detection filters, which prioritize genes with a localized (spatial or HD) expression patterns. The genes that exhibit a localized spatial or HD gradient are visualized in real time in the spatial domain, along with a number of options to enable user-tailored data exploration paths. This offers the user real-time querying of the gradient structure of ST data. In several use cases, we demonstrate that the spatial projection of these local kNN subgraphs highlights tissue architectural features and that interactive retrieval of gene expression gradients in the spatial and transcriptomic domains confirms known biology and provides additional insights into tissue architecture. We also show that SpaceWalker generalizes to several different ST protocols and scales well to large, multi-slice, 3D whole-brain ST data while maintaining real-time interaction performance.

## Results

### SpaceWalker overview

**Figure 1:**
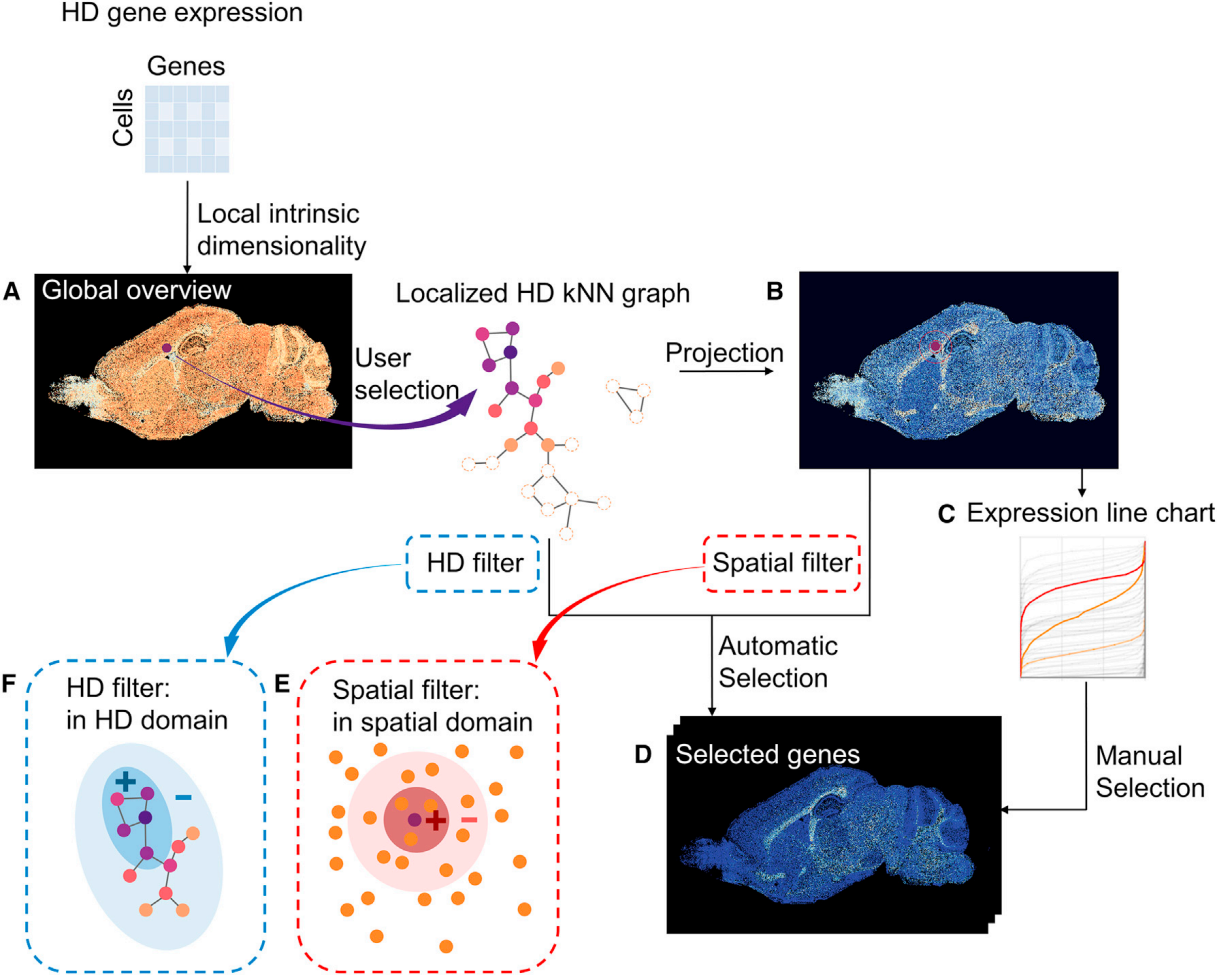
Overview of SpaceWalker The user interacts with a 2D scatterplot (A), where the spatial map is color-coded with the local intrinsic dimensionality of the HD data to highlight areas of potential interest. A flood-fill algorithm approximates the local topology of the HD space at the selected cell node and projects it back on the spatial map (B), displaying the HD structure and therefore transcriptomically similar cells. Genes with localized expression peaks are ranked automatically via a spatial (E) or HD filter kernel (F), and all genes are presented in a line chart (C) with their sorted expression values in the local neighborhood. The user can inspect the gene expression (D) of top-ranked genes (automatic selection) and can also manually select different genes of interest from the gene expression line chart (manual selection). Each line in the chart (C) represents a gene, and genes ranked the highest by the filter are highlighted in orange, and the manually selected gene is highlighted in red.

The goal of SpaceWalker is to provide the user with an interactive interface for exploring localized expression gradients in ST datasets. By offering highly responsive global and local linked views of cells and genes, SpaceWalker aims to help users identify tissue architecture as well as locally variable genes and to gain insights that would be difficult to uncover using script-based methods. An overview of the proposed methodology is shown in Figure 1. The input of SpaceWalker is a cell-by-gene expression matrix, with spatial coordinates assigned to the single-cell gene expression vector.^18^ A user’s exploration can optionally be guided by a global overview of local intrinsic dimensionality at each cell location (Figure 1A). This view can inform the user of potential transcriptomically complex locations in the spatial data. From a user-selected seed cell, the local HD structure is estimated and highlighted in the spatial map in real time, i.e., cells with similar transcriptomic profiles to the seed cell are presented to the user (Figure 1B). Genes with spatially localized expression peaks in the area around the selected cell are detected using a spatial filter kernel (Figure 1E) that ranks genes by localized expression variation. Alternatively, a filter for localized expression peaks in the HD space (Figure 1F) can be selected. These filters effectively serve as real-time gene image retrieval based on localized expression variability. Since multiple genes can exhibit similar filter responses, we also provide a line chart of all genes, showing sorted gene expressions in the local neighborhood (Figure 1C). This offers a complementary view to the filter gene rankings. The user can click on a gene of interest in the chart and inspect the gene expression of the chosen gene in a separate gene view (Figure 1D). This allows them to not only inspect the genes automatically selected by the filter but to also manually explore genes with interesting expression values in the local neighborhood.

### Real-time projection of the flood fill in the spatial domain reveals tissue architectural features

First, we investigated whether local tissue architectural features are reflected and preserved by projecting the localized HD neighborhoods on the spatial data. To this end, we used two publicly available ST datasets of the mouse visual cortex that were acquired in the context of the SpaceTx consortium.^19^ We selected these datasets (single-molecular fluorescence *in situ* hybridization [smFISH] and multiplexed error-robust FISH [MERFISH]) because of the abundance of literature on the laminar spatial architecture of the mouse visual cortex.^20^ The SpaceTx datasets contain manual annotation of the cortical layers, which enables investigating the robustness of SpaceWalker against ST protocols applied to the same tissue sample (smFISH and MERFISH).

**Figure 2:**
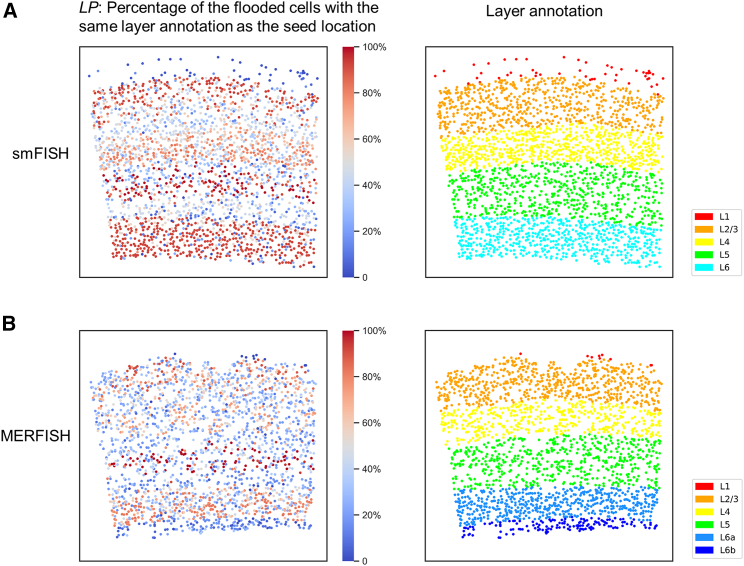
Spatial tissue maps color-coded by *LP* at each cell location and the manual layer annotation (A) smFISH dataset. (B) MERFISH dataset. *LP* is defined as the percentage of flooded cells with the same layer annotation as the seed cell. Note that the manual annotations are not used by SpaceWalker but only serve as a manual reference standard to compute the evaluation metric *LP*.

To quantitatively assess correspondence of the local neighborhood geometry with local tissue architecture, we defined a reference standard based on the known laminar structure of the mouse visual cortex. We defined the layer percentage (*LP*) as the percentage of flooded cells that have the same layer annotation as the seed cell: this assumes that transcriptomically similar cells are organized in spatial layers, which is the case for excitatory neurons in the visual cortex. For any selected cell, the projection of the local HD neighborhood reveals the spatial organization of cells with similar expression profiles (Video S1). Flooded neighborhood projections for every cell in the tissue clearly reveal that the flood-fill neighborhood searching recovers the laminar tissue architecture (Figure 2). Cells that are not located near the layer boundary on the spatial map show a high *LP* value, demonstrating that the local HD neighborhood projection accurately reveals the manually annotated laminar architecture of cells with similar expression profiles. Cells located near the boundary of the spatial layer are less likely to be in the same layer as the manual seed cell annotation and thus show a lower *LP* value. Another explanation for the lower *LP* values could be due to the fact that transcriptomically similar cells may appear in multiple layers.^20^ This cell-type cross-talk across cortical layer boundaries explains why flood-fill neighborhood searching seeded close to the manually annotated layer boundaries may span across the manually annotated layer boundaries. MERFISH shows overall lower *LP* values than smFISH due to factors such as scattered distribution of glutamatergic neurons and difference of gene counts.^20^ However, the SpaceWalker results obtained from the independently acquired smFISH and MERFISH datasets exhibit very similar layer patterning, as shown in Figure 2, indicating the robustness of SpaceWalker against the ST protocol and reproducible tissue architecture recovery across datasets.

Video S1. An exploration scenario with SpaceWalker using SpaceTx smFISH data, related to Figure 2

**Figure 3:**
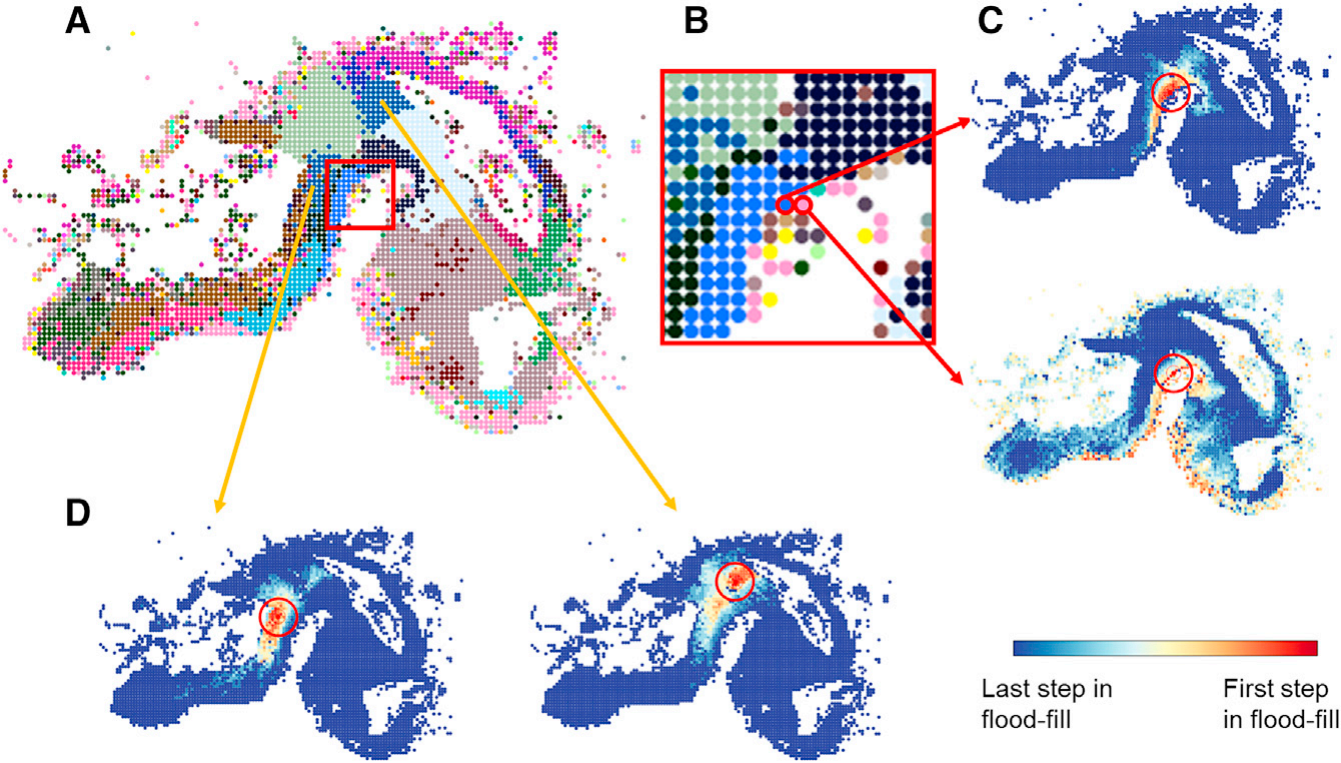
HD neighborhood projections by flood filling agree with subclass annotation in the HybISS data (A) Spatial map color-coded with cell subclass annotation. Note that the annotations are not used by SpaceWalker but only serve as a manual reference standard. (B) Magnified view of (A) showing two neighboring cells in the spatial map belonging to the hindbrain floor plate and mesenchyme, respectively. (C) HD neighborhood projections seeded from the two marked cells in (B), highlighting distinct spatial patterns that coincide with the region labels of hindbrain floor plate and mesenchyme. (D) HD neighborhood projections starting from two ventral hindbrain cells that are located in two different regions in the spatial map, highlighting the transcriptomic similarity between the two different ventral hindbrain regions. The selected seed cell in (C) and (D) is located in the center of the red circle, and the red circle represents the radius of the spatial filter. The coloring in (C) and (D) indicates the step index of flood fill, beginning with red points (initial steps) and ending with light blue points (late steps). Dark blue points represent the background, i.e., the cells that are not visited in flood fill.

To investigate whether SpaceWalker neighborhoods also reflect more complex spatial geometries accurately, we applied SpaceWalker to the HybISS data of the developing mouse brain.^21^ When projecting the local HD neighborhood back to the 2D spatial map, the cells are colored by their flood-fill step index, which represents a measure for the HD geodesic distance to the seed point. The neighborhood mapping presents similar patterns to the gold-standard region labels that were imputed from single-cell transcriptomics data^22^ (Figure 3A). For example, HD neighborhood projections highlighted the structure of the hindbrain floor plate and mesenchyme, even if the two starting cells of the flood-fill algorithm were located next to each other in the spatial map (Figures 3B and 3C). HD neighborhood projection also highlighted the similarity between ventral hindbrain cells that are not located close by in the spatial map (Figure 3D).

### Real-time gene filtering reveals localized gene expression peaks and gradients in the spatial domain and HD domain

To quantitatively evaluate to what extent the top-ranked genes detected by the spatial and HD filters corresponded to known layer-specific marker genes in the mouse visual cortex,^20^ we calculated a frequency count for filtered genes by counting how often genes end up in the top two ranks at different seed cell locations per cortical layer. It is important to note that the frequency counts by the filters are computed without using the layer annotations.

Apart from detection capacity for known marker genes, we aimed to assess whether SpaceWalker can also detect previously unreported genes that exhibit clear laminar expression patterns. As such, we defined a quantitative metric for the layer specificity of all genes by computing the correlation between gene expression images and the manual layer annotations. For each cortical layer, a layer mask was constructed based on the manual layer annotations, with cells within a specific layer encoded as 1 and all other cells encoded as 0. The correlation scores between all genes and the gold-standard layer masks are used as benchmark reference for the evaluation of filters. Finally, to assess robustness with respect to the ST protocol, we computed both metrics for the smFISH as well as the MERFISH data from the SpaceTx consortium. By comparing results of these two metrics, we aim to assess whether SpaceWalker can identify genes with layer patterns without any prior annotation and compare these results to the gold-standard manual layer annotation.

**Figure 4:**
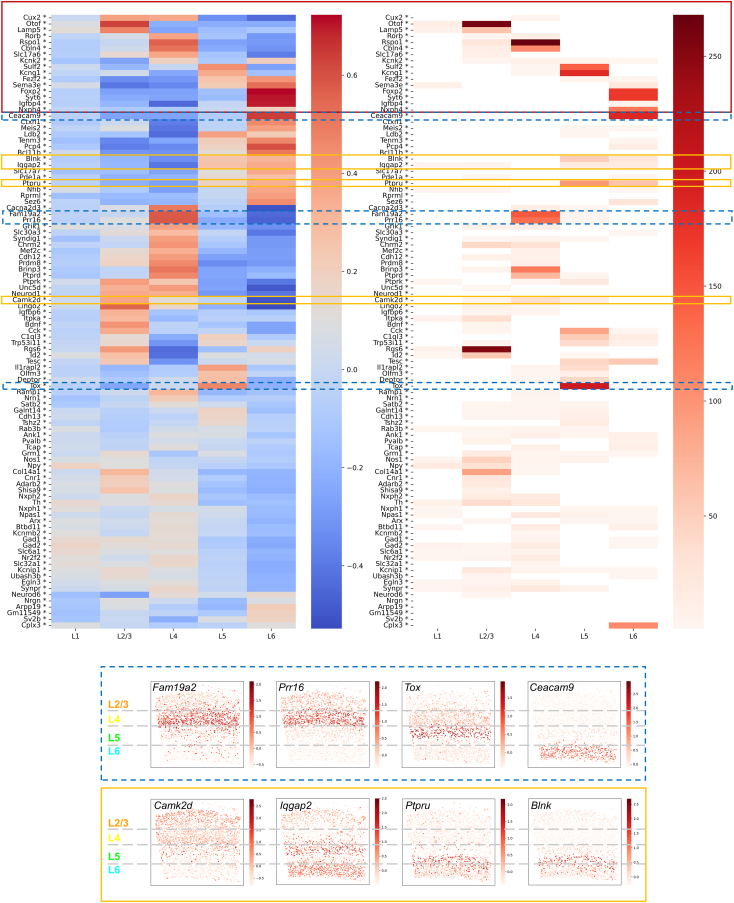
Heatmaps comparing the frequency counts by the spatial filter with the correlation scores between genes and layer annotation masks of the smFISH dataset Left: Heatmap of the correlation scores between genes and layer annotation masks. Right: Heatmap of frequency counts by the spatial filter. Known marker genes as reported^20^ are highlighted in the red box. Genes with a high correlation with a manually annotated layer mask were also often ranked in the top two by the spatial filter. Filter-detected genes that express in multiple layers are highlighted in yellow boxes. Examples of genes that are not reported in the literature as known layer markers but are frequently ranked as top two by the filter are shown in blue dashed boxes. Results of the MERFISH dataset are given in Figure S1. Results for the HD filter showed similar correspondence between correlation scores and frequency counts and are given in Figures S2 and S3.

The SpaceTx smFISH dataset consists of 2,360 cells and 314 genes. To reduce the impact of genes with noisy patterns, we refined this dataset by filtering down to 100 top high-variance genes (HVGs). The 100 top HVGs formed the basis for the analysis presented in Figure 4. The variance-sorting function is also integrated in SpaceWalker, allowing the user to conveniently filter genes according to variance. Figure 4 shows a side-by-side comparison of the heatmaps of the correlation scores and the frequency counts of the filtered genes of the smFISH data (results of the MERFISH data are given in Figure S1). All layer-specific marker genes have a high correlation score with their corresponding layer annotation, proving that the correlation metric identifies genes with contrasting patterns within a specific layer. Known marker genes as reported^20^ are presented at the top of the heatmaps, and the other genes are grouped based on layer and correlation score. Genes with a high frequency count in a specific layer also exhibit a high correlation score with the manually annotated layer masks, demonstrating the ability of the filters to capture local spatial variability. Some genes with a high ranking in our filters are not mentioned in the literature as known layer markers even though they were highly correlated with the manually annotated layer masks. Visual inspection of these genes revealed clear laminar expression patterns.

It is important to note that the filter results do not perfectly align with layer-specific analysis results, as the spatial filter is designed to be universal in its use and to identify genes with local variability within the filter kernel, while layer-specific analysis is contrasting the expression vectors inside and outside of a layer. The filter size used in the comparison with layer-specific analysis is approximately equal to the spatial size of the layer, providing a basis for comparison. However, the user can adjust the filter size based on their interests to define the locality of the gene expression patterns (Video S3).

As the filters only take cells within the filter kernel size into account, it is possible that layer-structured genes are detected that are also highly expressed in other locations. It can also be seen in the heatmaps in Figures 4 and S1 that a gene can be ranked as top two in multiple layers if it has high correlation scores with multiple layers.

### Color-coding the spatial map with localized HD features provides visual cues for guidance of exploration

**Figure 5:**
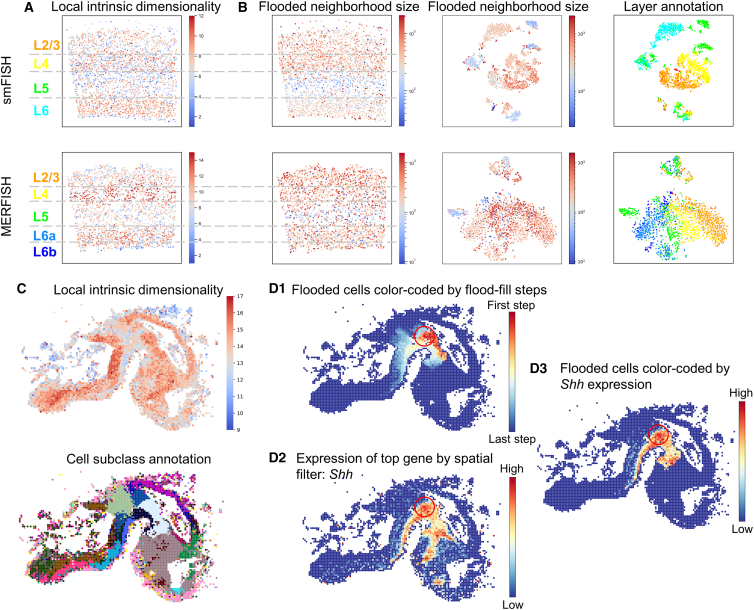
Spatial maps color-coded by localized HD features (A) Spatial maps color-coded by local intrinsic dimensionality of smFISH and MERFISH datasets. (B) Spatial maps color-coded by flooded neighborhood size (left) and t-SNE maps color-coded by flooded neighborhood size (middle) and layer annotation (right) of smFISH and MERFISH datasets. (C) Spatial maps of the HybISS dataset, color-coded by local intrinsic dimensionality and cell subclass annotations. (D1–D3) Color-coding of flooded HD neighbors by flood-fill index (D1) and *Shh* expression (D3), where *Shh* was ranked as the top gene by spatial filtering at the defined location. The selected seed cell is located in the center of the red circle, and the red circle represents the radius of the spatial filter. The entire spatial map colored by *Shh* expression is shown in (D2) as a reference.

Figures 5A and 5B give examples of spatial maps in the SpaceTx datasets, color-coded by HD intrinsic dimensionality and flood-fill size. The laminar structure clearly emerges from the intrinsic-dimensionality coloring (consistent in both smFISH and MERFISH datasets), indicating that local intrinsic dimensionality provides visual cues for spatial tissue partitioning. The local intrinsic dimensionality of layer 6 (L6) is found to be higher than other layers, while the locally explained variance of L6 is lower. This suggests that the cells in L6 are less homogeneous than cells in other layers. L5 has a relatively dense HD structure compared to other layers, where the flood fill is confined within a dense cluster. In contrast, the flood fill progresses further in layers with relatively sparse HD structures, leading to a larger neighborhood size. This can be confirmed by t-SNE maps also color-coded by flood-fill size where cells in L5 are divided into multiple smaller clusters, while the cells in the other layers form distinct and well-separated clusters (Figure 5B).

Figure 5C shows how the intrinsic dimensionality can serve to guide the user to anatomically distinct regions in the HybISS data, demonstrating that changes in local intrinsic dimensionality, in many cases, mirror transitions between cell subclasses. Color-coding of the flood-fill cells by flood-fill step index (Figure 5D1) reveals the local HD topology on the spatial map, whereas color-coding by gene expression of the top-ranked *Shh* gene (Figure 5D3) reveals spatially localized gene expression.

### SpaceWalker scales to 3D whole-mouse brain ST at real-time interaction speeds

In the above sections, we have used two SpaceTx datasets and a HybISS dataset to validate the functionality of SpaceWalker. The samples of the mouse visual cortex in the SpaceTx datasets have a well-characterized tissue architecture and feature structure, demonstrating real-time performance on these datasets on consumer-grade computing platforms. These datasets are relatively small in scale (Table S1). Scalability in biological data analysis is considered of significant importance due to the increasing complexity of data^13^^,^^17^^,^^18^ as laboratory technologies continue to evolve. ST protocols such as enhanced electric (EEL) FISH^18^ now scale toward large field of view (FoV) whole-organ tissue patches. Alternatively, through stitching of multiple patches, large tissue surface areas can now be compounded to a large FoV.

To investigate whether SpaceWalker still facilitates real-time exploration at such large patches, we deployed it to a sagittal whole-brain slice acquired with EEL FISH.^18^ This dataset consists of 127,591 cells and 440 genes. The user can then start interacting smoothly with the interface based on real-time computation. An overview of computation times and memory footprints for all experiments in this article are given in Table S1.

**Figure 6:**
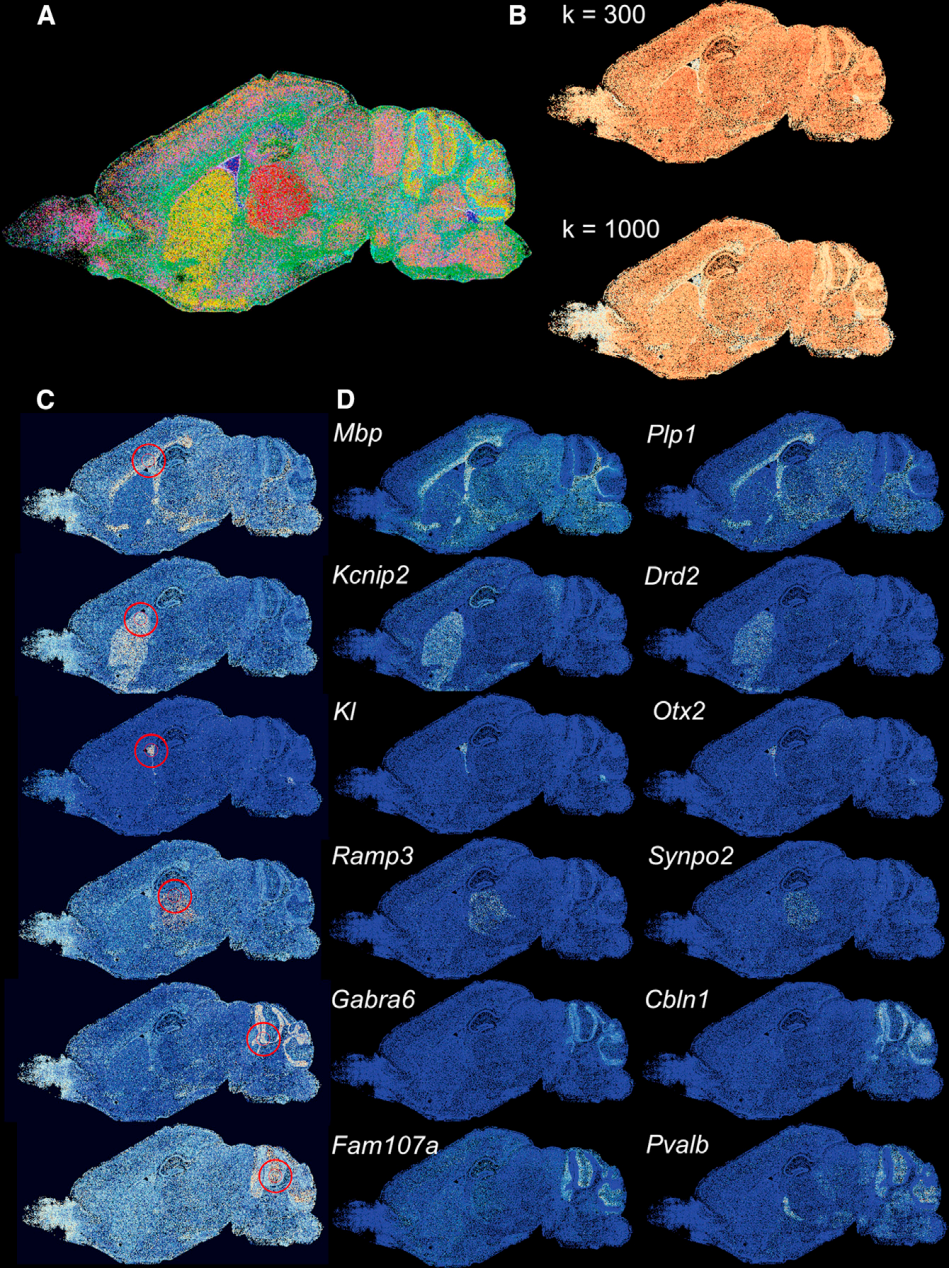
Exploration results of the EEL FISH dataset revealing tissue structure and local gene patterns (A) Reference annotation, in which 30 selected genes highlight anatomical structures of a sagittal mouse brain section.^18^ (B) Spatial maps color-coded with local intrinsic dimensionality with k = 300 and 1,000, indicating that local intrinsic dimensionality provides visual cues about region boundaries. (C) Examples of local neighborhood reprojections. Flood-fill cells are colored by flood-fill step index from red to light color and background cells with dark blue. The selected seed cell is located in the center of the red circle, and the red circle represents the radius of the spatial filter. (D) Top two genes selected by spatial filtering at the location of the seed cell in (C), each highlighting spatial gene expression that is similar to the flood-fill geometry.

Next, we investigated whether SpaceWalker exploration of this large dataset maintained similar tissue architecture retrieval and feature detection performance as with the smaller datasets reported above. Figure 6 gives examples of local intrinsic dimensionality based on two different kNN graphs, local flood-fill reprojections, and identification of genes with localized expression patterns detected by the spatial filter. Cells in the cerebellum, corpus callosum, and olfactory bulb exhibit lower intrinsic dimensionality than other tissue areas, delineating anatomical region boundaries (Figure 6B). The local neighborhood projections (Figure 6C) highlighted the anatomical regions in EEL FISH and identified genes whose spatial expression profiles resembled the flood-fill geometry (Figure 6D). Also, several of the filter-ranked genes were detected that corresponded to genes reported^18^ (*Mbp* and *Plp1* in the corpus callosum; *Drd2* in the striatum; *KI* and *Otx2* in the choroid plexus; *Ramp3* and *Synpo2* in the thalamus; and *Cbln1* and *Fam107a* in the cerebellum). These results demonstrate that SpaceWalker scales to large, whole-slide ST data while maintaining interactive speed (Video S2) and still retrieves genes that confirm known local tissue biology.

Video S2. SpaceWalker scales to whole-mouse brain section at real-time performance using EEL FISH data, related to Figure 6

Video S3. The interactive selection in SpaceWalker using HybISS data, related to Figures 3 and 5

As we demonstrate above, SpaceWalker is capable of exploring large-scale ST tissue sections at interactive performance. SpaceWalker is not restricted to handling one single spatial slice; it also supports multi-slice exploration. With the recent emergence of whole-brain ST atlases,^23^ ST data can be visualized and explored in a multi-slice manner and in a whole-brain 3D common coordinate frame. To investigate the scalability of SpaceWalker toward 3D whole-brain ST data, we deployed SpaceWalker on the Allen Brain Cell (ABC) Atlas^23^: a 3D-annotated whole-brain ST dataset of the mouse brain consisting of ∼4 million cells, all mapped to a common 3D coordinate frame.

For this specific 3D dataset, we implemented a dedicated 3D volume renderer view to visualize the flood-fill geometry and gene filtering results in 3D, as well as a multi-slice browser view that allows smooth scrolling through the slices and applies the localized gene filtering. The flood fill is performed on all cells in the 3D atlas. The flood-fill results can then be viewed on multiple slices by scrolling, as well as in the 3D common coordinate frame. Gene filtering is performed on individual slices, and the user can simply scroll through the 2D slices while inspecting the detected gradient genes in 3D.

**Figure 7:**
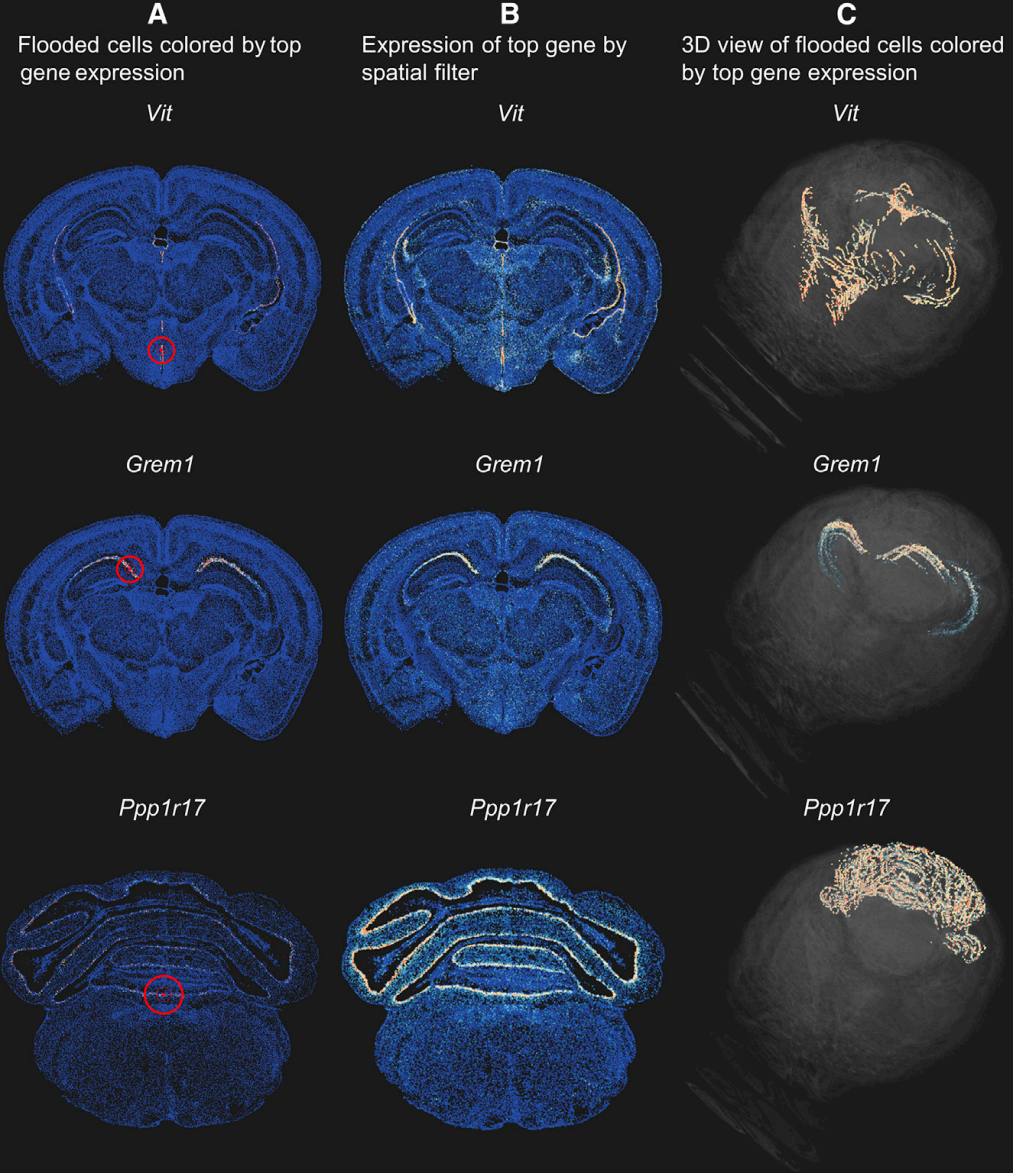
Results of 3D whole-brain multi-slice exploration of the ABC Atlas dataset (A) Local neighborhood reprojections. Flooded cells are colored by the expression of the top gene identified by spatial filtering at the specified location. Selected seed cell is marked with a red dot in the center of a red circle representing the outer radius of the spatial filter. (B) Spatial maps of the brain slice, colored by the expression of the top gene, as a reference. (C) 3D views of flooded cells, colored by the expression of the top gene. The results are derived from brain sections 25 (top and middle row) and 0 (bottom row). A red-blue color map is used to indicate gene expression levels, with red representing a high expression value and blue representing a low expression value. More examples are given in Figures S4 and S5 and Video S4.

We applied SpaceWalker to 3.7 million cells labeled as high quality in the ABC Atlas and to the full feature set (550 genes). SpaceWalker is capable of identifying 3D tissue structures as well as localized gene expression patterns while remaining performant and fully interactive. Symmetric tissue structures are highlighted by flood filling when the seed cell located in one part of the spatial map and related tissue structures across slices are highlighted in 3D during exploration on one single slice. Examples of the exploration results are given in Figures 7, S4, and S5. The 3D viewing feature and the interactive performance are demonstrated in Video S4.

Video S4. SpaceWalker scales to 3D whole-mouse brain using Allen Brain Cell Atlas data, related to Figure 7

## Discussion

Interpretation and analysis of ST datasets is often limited to script-based tooling with limited interactivity. However, hypothesis generation from complex biological data benefits from interactive, on-the-fly querying of the data, especially for ST data, since genes can exhibit different functions at different locations in the tissue. This also holds for localized spatial or transcriptomic expression gradients: spatial expression gradients are important indicators for tissue boundaries, migration trajectories, and spatially evolving cell phenotypes, whereas transcriptomic gradients reflect cell states, differentiation, and cell-type boundaries.

Here, we present SpaceWalker, an interactive visual analytics tool for exploration of large patches of ST data. SpaceWalker consists of two key innovations: an interactive, real-time flood fill and spatial projection of the local topology of the HD space, and a gradient gene detector for on-the-fly retrieval of locally variable genes from the full gene set. We complement this with a suite of user-defined visualization options to inspect and query the data and to project analysis results on the spatial data.

Compared with existing approaches, SpaceWalker is more flexible for data exploration, especially when no prior knowledge regarding the tissue structure and gene expression patterns is available. First, SpaceWalker introduces an interactive, real-time flood-fill algorithm to reveal tissue structures. This differs from existing approaches, such as HMRF,^7^ SC-MEB,^8^ and BayesSpace,^9^ which focus on cell clustering within the entire dataset. Instead, SpaceWalker focuses on a user-defined, size-adjustable local neighborhood, providing a more flexible exploration of the tissue structure. Secondly, SpaceWalker features on-the-fly gene filters for locally variable genes. Unlike these existing clustering approaches, where differential expression analysis is often followed to identify marker genes related to the identified clusters, SpaceWalker does not require prior clustering for gene retrieval. The gene retrieval in SpaceWalker is based on user-defined local neighborhoods, and the selection of the neighborhood is easy to adjust and is interactive. Other spatial analysis algorithms, such as SpatialDE^10^ and Trendsceek,^11^ are capable of identifying genes with spatial expression patterns. However, these techniques are script based and lack the capacity to detect localized gene expression patterns at user-defined cell locations—a feature that SpaceWalker incorporates. Toolboxes like Giotto,^12^ squidpy,^13^ CELLxGENE,^17^ and stLearn,^24^ provide interactive viewers for data exploration, but their integrated analysis algorithms suffer from similar limitations due to their script-based nature. Unlike the aforementioned tools and algorithms, which are designed to identify gene expression patterns based on the entire input dataset, SpaceWalker is capable of identifying genes with localized gene expression patterns during interactive exploration. In summary, we propose a novel exploration tool for spatial neighborhood projection and real-time retrieval for gene expression patterns. The comparison between existing approaches and SpaceWalker is given in Table S2.

We quantitatively validated SpaceWalker results on public datasets of varying data sizes, demonstrating that SpaceWalker accurately captures tissue architectural features while at the same time retrieving locally expressed genes, in many cases coinciding with known marker genes. SpaceWalker scales very well to large datasets: only the pre-processing time increased, while on-the-fly exploration speed remained near constant for datasets ranging from 4,628 cells × 117 features to 3.7 million cells × 550 features. Taken together, these results demonstrate that SpaceWalker enables linked visual exploration of tissue architecture and (spatial and HD) gene expression gradients in 2D and 3D. It faithfully recapitulates known tissue architecture and biology in an interactive manner in 2D ST datasets but also offers interactive facilities to recover localized expression gradients in 3D whole-brain ST data, for instance in the midbrain, where cell-type distributions have been shown to exhibit gradient patterns.

### Limitations of the study

The use of distance metrics to compute neighborhoods in HD space in SpaceWalker may present a challenge, known as the curse of dimensionality, when the dimensionality of the datasets reaches a certain size. We tested SpaceWalker with a feature depth of maximum 550 genes in ABC Atlas data. Datasets with a higher dimensionality may have less clear separation between the data points due to this challenge. In such datasets (for instance where the spatial data are imputed with transcriptome-wide gene expression from scRNA-seq data), the HD nearest neighbors could be computed based on a principal component analysis (PCA) projection of the full data. The spatial gene filtering could still be performed over the full imputed feature set, enabling transcriptome-wide exploration of gradients over all genes.

As previously mentioned, SpaceWalker can also be applied to data of the developing brain and revealed tissue-structure-related patterns. Though out of scope for this work, it would be interesting to further investigate SpaceWalker’s performance on multi-time point developmental datasets as well as extend SpaceWalker for exploring developmental trajectories. Moreover, while we have focused on the spatial map, the spatial filters can also be applied to any 2D maps, such as t-SNE and uniform manifold approximation and projection (UMAP)^25^ embeddings. For example, the spatial filters can be applied to a t-SNE map with a size approximately corresponding to the spatial size of subclass clusters to identify subclass-specific genes or expression gradients within or between clusters in t-SNE and UMAP scatterplots.

Overall, SpaceWalker offers a novel and interactive visual interface for exploring ST data. By visually presenting the HD neighborhood structure of cells and genes with localized expression patterns, SpaceWalker helps study the tissue structure and identify potential genes of interest. SpaceWalker focuses on retrieving expression gradients and HD geometry on the fly, which differentiates it from the current state of the art in script-based ST tooling and is therefore complementary in its application and not a suggested replacement.

## STAR★Methods

### Key resources table

**Table:** 

REAGENT or RESOURCE	SOURCE	IDENTIFIER
**Deposited data**		

SpaceTx	Long et al.^19^	https://viewer.cytosplore.org
HybISS	La Manno et al.^21^	http://mousebrain.org/development/downloads.html
EEL FISH (the Mouse 440 gene RNA data)	Borm et al.^18^	http://mousebrain.org/adult/downloads.html
Allen Brain Cell Atlas	Yao et al.^23^	https://github.com/AllenInstitute/abc_atlas_access/blob/main/descriptions/MERFISH-C57BL6J-638850.md

**Software and algorithms**		

SpaceWalker	This paper	https://zenodo.org/records/10017490

### Resource availability

#### Lead contact

Further information and requests for resources and reagents should be directed to and will be fulfilled by the lead contact, Boudewijn Lelieveldt (b.p.f.lelieveldt@lumc.nl).

#### Materials availability

This study did not generate new unique reagents.

#### Data and code availability

• This paper analyzes existing, publicly available data. Links for the datasets are listed in the key resources table.
• SpaceWalker is implemented in C++ as a plugin of the ManiVault plugin system^26^ for visual analytics application building. All original code has been deposited at https://github.com/ManiVaultStudio/SpaceWalker and is publicly available as of the date of publication. DOIs are listed in the key resources table. The Windows installer for SpaceWalker and system state files containing the data, plugins and GUI configurations are available at https://github.com/ManiVaultStudio/SpaceWalker. Installers for MacOS and Linux will be made available at www.cytosplore.org in the future.
• Any additional information required to reanalyze the data reported in this paper is available from the lead contact upon request.

### Method details

#### Data

All data was standardized and subsequently scaled to a range between 0 and 1 using min-max normalization when loaded into SpaceWalker.

SpaceTx datasets were downloaded from https://viewer.cytosplore.org. We used the imputed dataset, where smFISH and MERFISH datasets have the same number of genes. Genes were prefiltered to top 100 highly variable genes before SpaceWalker analysis in Figures 4 and S1–S3. All 314 genes were used in other analyses presented in this paper (Figure 2; Table S1).

HybISS data was downloaded from http://mousebrain.org/development/downloads.html. The original HybISS data was preprocessed and imputed, and the annotations were transferred from single-cell transcriptomics to the spatial SIRV data by Abdelaal et al.^22^ EEL FISH data was downloaded from http://mousebrain.org/adult/downloads.html (the Mouse 440 gene RNA data). ABC Atlas data was downloaded from https://github.com/AllenInstitute/abc_atlas_access/blob/main/descriptions/MERFISH-C57BL6J-638850.md. The sizes of the utilized datasets are shown in Table S1.

#### Flood-fill projection

Before interactive exploration of the spatial map with flood-fill projections, an HD neighborhood graph is either computed (using FAISS^27^) or loaded from a file, which stores the exact nearest neighbors of every data point. Multiple metrics can be used when computing this graph, including Manhattan distance, angular distance, as well as secondary similarity measures^28^ to improve quality on higher-dimensional datasets.

During exploration, when the user selects a seed cell in the spatial map, its k direct neighbors are added to the flood-fill. Then, for n steps, the k direct neighbors of the cells added in the previous step are added to the flood-fill. Neighbors that have already been added are not added again. Both k and n are interactive user-defined parameters that change respectively how broad and how deep the flood-fill goes. Importantly, this process differs from simply computing the seed cell’s nearest neighbors with a higher k value, as each wave of the flood is restricted to a local neighborhood graph. This makes it improbable that a flood-fill wave spreads to dissimilar cells as those are unlikely to be part of the local neighborhood. On the other hand, computing the cell’s nearest neighbors with a large k value can easily cross to dissimilar clusters in the HD space if the size of k exceeds that of the local cluster. Therefore, the flood-fill algorithm more closely approximates the neighborhood of similar cells and extinguishes itself when it reaches the boundaries of that neighborhood.

Figure S6 uses a simplified example to demonstrate the difference between the neighbors selected by the flood-fill algorithm and direct nearest neighbors. The flood-fill only proceeds within the cluster, while the direct nearest neighbors also include nodes from a separate cluster.

#### Whole-slide visualization of local HD intrinsic dimensionality

The visual exploration in SpaceWalker can be guided by the number of features that express the local variance of the HD gene expression space, as a proxy for biological variability (Figure 1A). For every cell, we compute the local intrinsic dimensionality in a defined HD neighborhood, which is then used to color-code the spatial map.

The term 'local intrinsic dimensionality' refers to the minimum number of dimensions required to represent a pre-set fraction of the data variance. Considering that certain genes can be highly correlated, the data can be represented using fewer dimensions without losing much information. Therefore, we define 'local intrinsic dimensionality' as the number of dimensions needed to account for 85% of variance within the local subset of data. This subset of data corresponds to the neighborhood of a specific cell. More specifically, we apply PCA^29^ on an n-by-m matrix at each cell location, where n is the number of neighbors of a specific cell and m is the total number of genes. Every cell point is color-coded by the number of principal components required to model 85% of the total local variance.

By presenting the local intrinsic dimensionality at each cell location, our intention is to provide an overview of local data complexity at each cell location in the tissue. This provides a global overview of region boundaries to guide the spatial exploration by transcriptomic variability.

In addition to features expressing local variance, we also provide exploration guidance in the form of a metric for local density of the HD space. The flood-fill algorithm used to identify the nearest neighbors of a seed cell is controlled by a fixed number of steps, resulting in varying flood-fill sizes at each seed cell location. The flood-fill size reflects the HD density of the dataset, with a larger neighborhood indicating a sparser HD structure and a smaller neighborhood indicating a denser structure.

#### Localized gene filtering and selection

ST data is often used to explore boundaries between transcriptomically distinct tissue regions and cell mixtures, differentiation trajectories or cell migration paths. Such patterns are typically characterized by localized changes in the expression of one or more genes. To enable the study of such localized gene expression gradients, we developed an exploration mode that enables the user to interact on the 2D map, while at the same time genes with significant localized expression patterns are ranked by three filters: 1) a spatial peak filter (Figure 1E), representing differential expression between the average gene expression vectors of two 2D circular spatial neighborhoods with different radii: such filters contrasting two different spatial neighborhood sizes are common in classical image processing^30^; 2) an HD filter (Figure 1F), contrasting the average gene expression vectors within two flooded HD neighborhoods of different number of steps; 3) the third option is to apply the spatial peak filter only on the (transcriptomically similar) flooded cells or on cell subtypes selected from a cell-type taxonomy, this enables the exploration of spatial expression gradients within isolated cell subtypes (e.g., glutamatergic neurons) without mixing of cell types in the gene filter region of interest. We opted for peak filtering by computing differential expression between a small and a large (spatial or HD) neighborhood due to its low computational complexity and rotational invariance in the spatial domain. Additionally, peak filtering has the capacity to detect both peaks and gradients with the same filter, since the gradient is often near the peak, reflecting the transition from high to low values. The filter neighborhood sizes can be modified through interactive sliders on the user interface, where the ratio between neighborhoods shifts the filter properties between peaks and gradients.

The aforementioned filters enable an automated detection of genes with localized expression patterns by sorting genes according to filter response. The top-ranked genes are presented in linked panels for their spatial expression patterns, and can be inspected by clicking on the gene panel of interest (Videos S1 and S2). However, co-expressing genes may have slightly lower filter ranks, therefore we enable the user to interactively deviate from the automated gene selection based on ranks. In a line chart, we plot a series of lines, where each line represents the sorted (low-to-high) gene expressions of that particular gene over the flooded cells (Figure 1C). Genes with a high-ranked filter response are highlighted in orange on the chart. While the user explores different cell locations on the map, gene expression values of the flooded cells are updated in the line chart, providing an overview of local gene expression patterns. The user can interactively select a line in this chart to see the spatial gene expression map of the corresponding gene. This enables the user to investigate other genes of interest based on their gene expression profiles or prior knowledge. For example, a gene showing high expression values throughout all of the cells within the flood-fill might indicate a large contribution to the flooded neighborhood. Alternatively, a gene showing a gradual change in gene expression could point to cell maturation trajectories.

#### Coloring flood-fills by localized features

Flooded cells can be colored with different features, i.e., foreground cells are highlighted, and colored with a different feature than the background cells (Figure 5 (d1) (d3)). These localized color-coding options assist the user in exploring the local subspace properties in the context of HD geodesic distances and expression gradients. The user can then interact with the map and explore local data properties by selecting one of the following flood-fill color-codings.

• HD geodesic distance approximation: Flooded cells are color-coded by their flood-fill step index to indicate their proximity to the seed cell in HD space, hence reflecting local HD kNN graph geometry. The user can interactively define the number of flood-fill steps to inspect the HD topology.
• Gene expression: Flooded cells are colored with the expression of the top ranked gene, and all other cells with a constant background color. The user can also manually select other genes of interest from the gene expression line chart for coloring the flooded cells. The localized view of gene expression enables the user to visually evaluate the spatial geometry of the flood-fill in combination with the gene expression pattern.

#### Parameter selection

SpaceWalker offers a highly interactive user interface, allowing easy modification of parameters throughout the exploration process, and on-the-fly visual inspection of the effect of parameter changes. However, it should be noted that variations in parameter selection may yield different results.

For the choice of distance metrics for computing local intrinsic dimensionality and flood-fill, we suggest using a combination of Manhattan distance with shared distances^28^ for smaller-depth datasets (containing fewer than 200 genes), as it tends to provide more accurate neighborhood results but it is computationally expensive. For datasets with larger feature depth, angular distance is a default and suitable choice. In SpaceWalker, the default distance metric depends on the number of dimensions of the dataset. The choice of neighborhood size affects how localized the local intrinsic dimensionality plot shows and the default is set as 30.

The flood-fill is controlled by the number of cells added at each flood wave (k) and the number of flood steps. A small k will result in a less branching exploration of the HD space, while a large k will branch out more. A small number of steps corresponds to a more local neighborhood, while a large number of steps will explore a larger neighborhood. The optimal parameters depend on the desired level of localization that the user expects for the revealed patterns.

The choice of filters may result in different top genes. The spatial filter without restriction to the flooded nodes is purely based on the spatial map and thus identifies the spatial expression patterns. The spatial filter with restriction to the flooded nodes reduces the effect of mixed cell types in the spatial region and identifies the spatial expression patterns in the transcriptomically similar cells. The HD filter is not restricted by the spatial coordinates, thus it identifies the gene expression patterns in the HD space.

For the spatial filter, the radii depend on both the size of the spatial map and the specific local patterns that the user expects to explore. Employing a small inner radius is beneficial for exploring more localized gene expression patterns, while larger inner and outer radii can identify larger expression patterns. For instance, in our study, we used a small spatial filter size for EEL FISH (Figure 6 and Video S2) to identify genes expressed within small anatomical structures. Conversely, larger radii were applied for SpaceTx datasets to identify genes that correspond to large layer structures (Video S1). The parameter for the HD filter follows a similar principle.

In summary, parameter selection with SpaceWalker depends on the dataset and the expected level of localization during exploration. However, the strong point of SpaceWalker is that the effect of parameter changes can be visually inspected and assessed on the fly, in contrast to script-based methods. An example scenario of interactive parameter selection and the difference of results is demonstrated in Video S3 using the HybISS data.

The default parameters and the specific parameters utilized in the analysis across all datasets in this work are given in Table S3.

### Quantification and statistical analysis

We used Pearson correlation as a reference for evaluation of marker gene identification in Figures 4 and S1.

## Supplemental information

Document S1. Figures S1–S6 and Tables S1–S3

Document S2. Article plus supplemental information
