# Supplementary material for: SpaceWalker enables interactive gradient exploration for spatial transcriptomics data
Source: Cell Rep Methods. 2023 Nov 15;3(12):100645. doi: 10.1016/j.crmeth.2023.100645 (PMC10753200; doi:10.1016/j.crmeth.2023.100645)
Supplement: Document S1. Figures S1–S6 and Tables S1–S3 [file mmc1.pdf]

Cell Reports Methods, Volume *3*

Supplemental information

SpaceWalker enables interactive gradient exploration for spatial transcriptomics data

Chang Li, Julian Thijssen, Thomas Kroes, Mitchell de Boer, Tamim Abdelaal, Thomas Höllt, and Boudewijn Lelieveldt

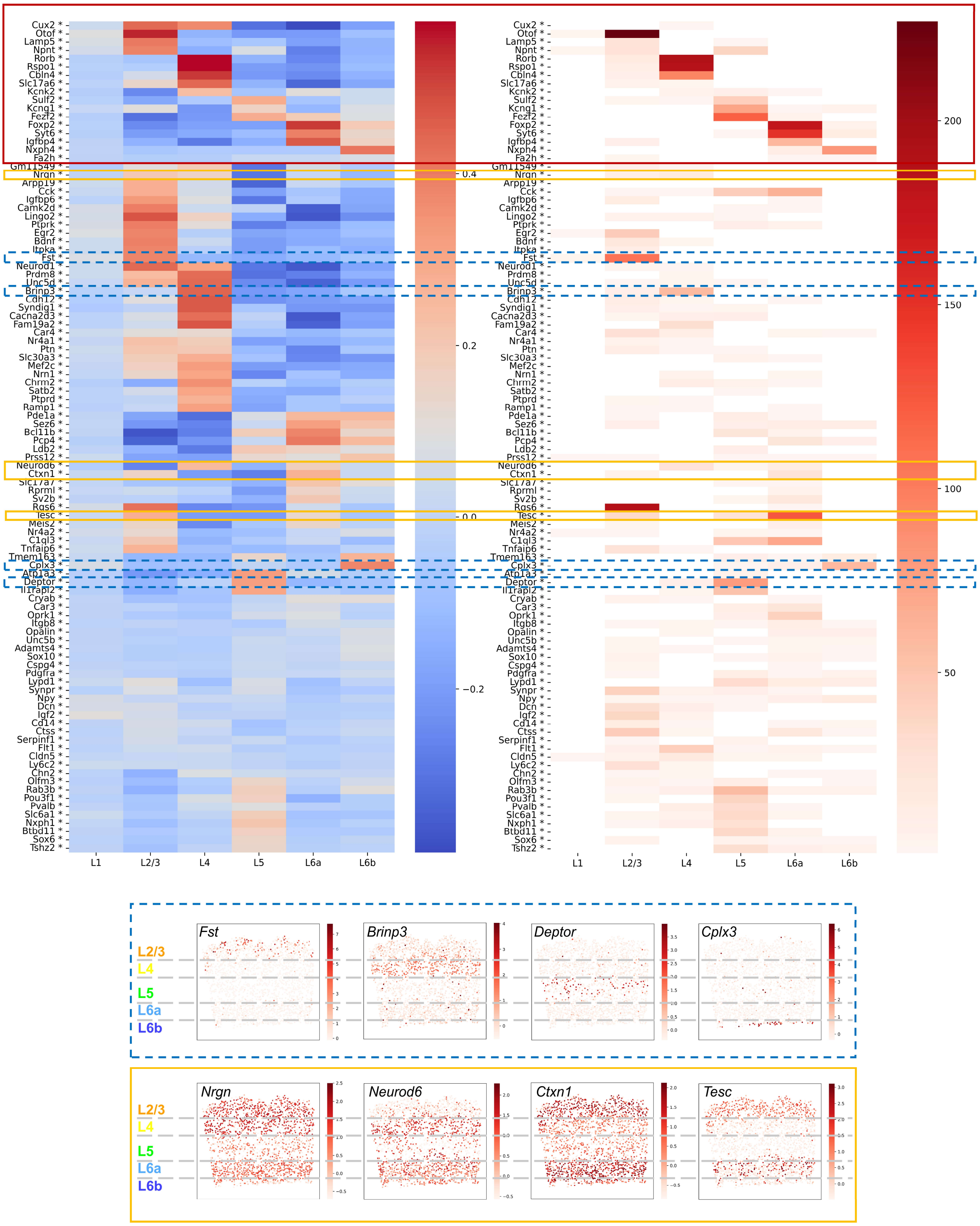

Supplementary Figure 1: Heatmaps comparing the frequency counts by the spatial filter (right) with the correlation scores between genes and layer annotation masks (left) of the MERFISH dataset, related to Figure 4. Known marker genes as reported[20] are highlighted in the red box. Genes with a high correlation with a layer mask were also often ranked in the top two by the spatial filter. Examples of genes that are not mentioned in the literature as known layer markers, but frequently ranked as top two by the filter, are marked in blue dashed boxes. Filter-detected genes that express in multiple layers are highlighted in yellow boxes.

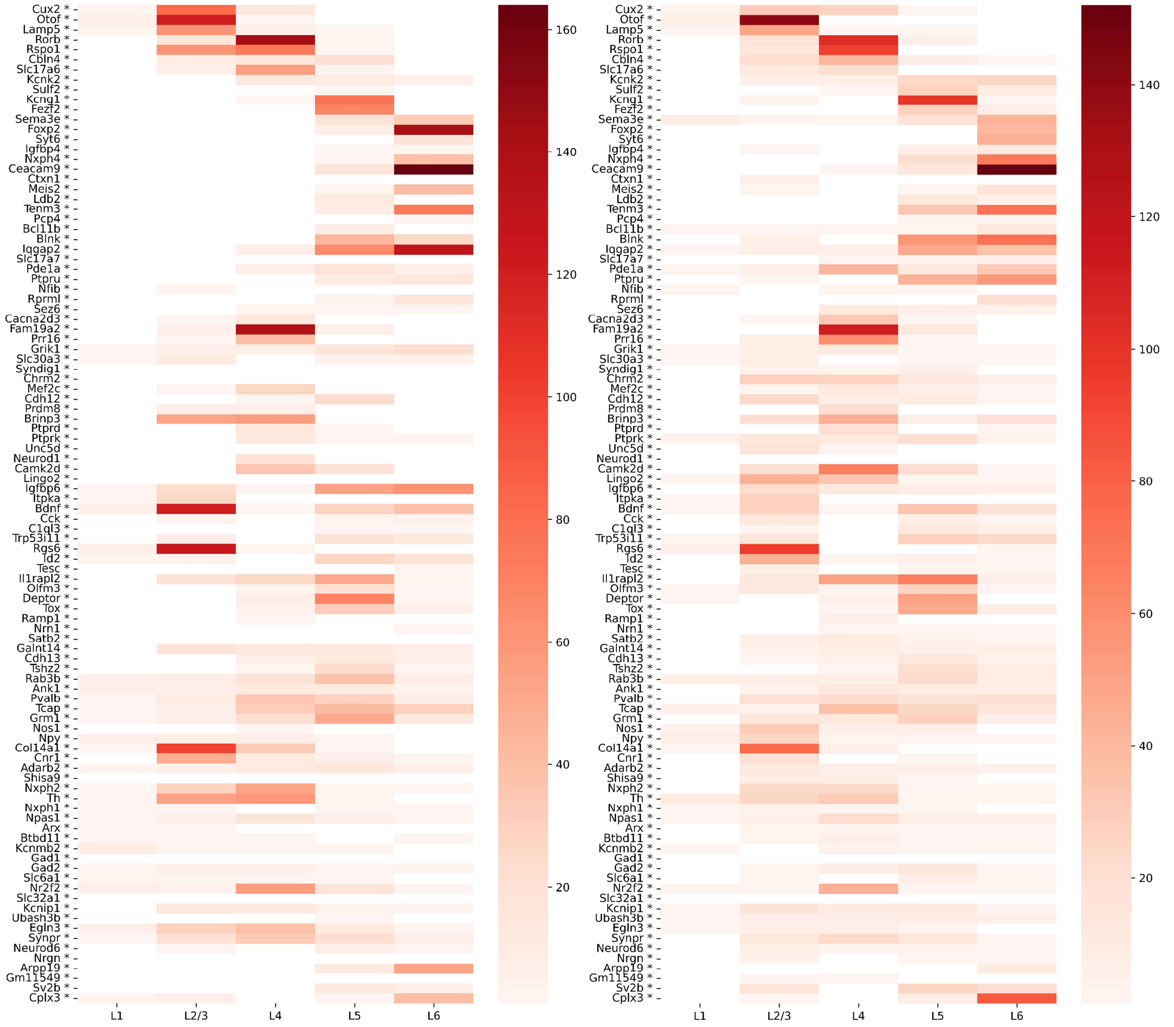

Supplementary Figure 2: Heatmap of the frequency counts by the HD filter in transcriptomics space (left) and heatmap of the frequency counts by the localized spatial filter that only applied on flooded cells (right) of the smFISH dataset, related to Figure 4. Similar to the results of the spatial filter (applied on all cells) in Figure 4, genes with a high correlation with a layer mask were also often top-two ranked by the HD filter and localized spatial filter.

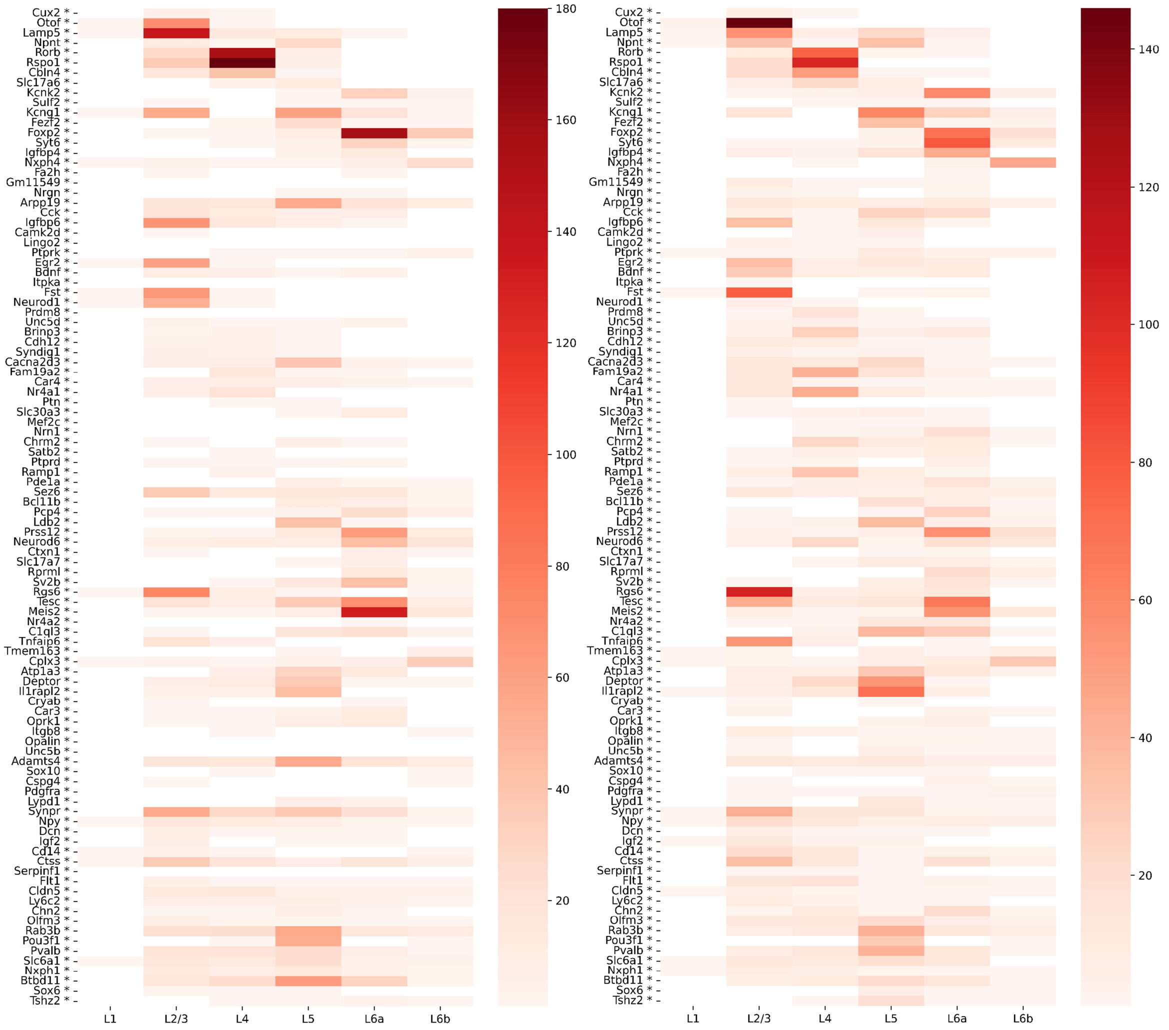

Supplementary Figure 3: Heatmap of the frequency counts by the HD filter in transcriptomics space (left) and heatmap of the frequency counts by the localized spatial filter that only applied on flooded cells (right) of the MERFISH dataset, related to Figure 4. Similar to the results of the spatial filter (applied on all cells) in Supplementary Figure 1, genes with a high correlation with a layer mask were also often top-two ranked by the HD filter and localized spatial filter.

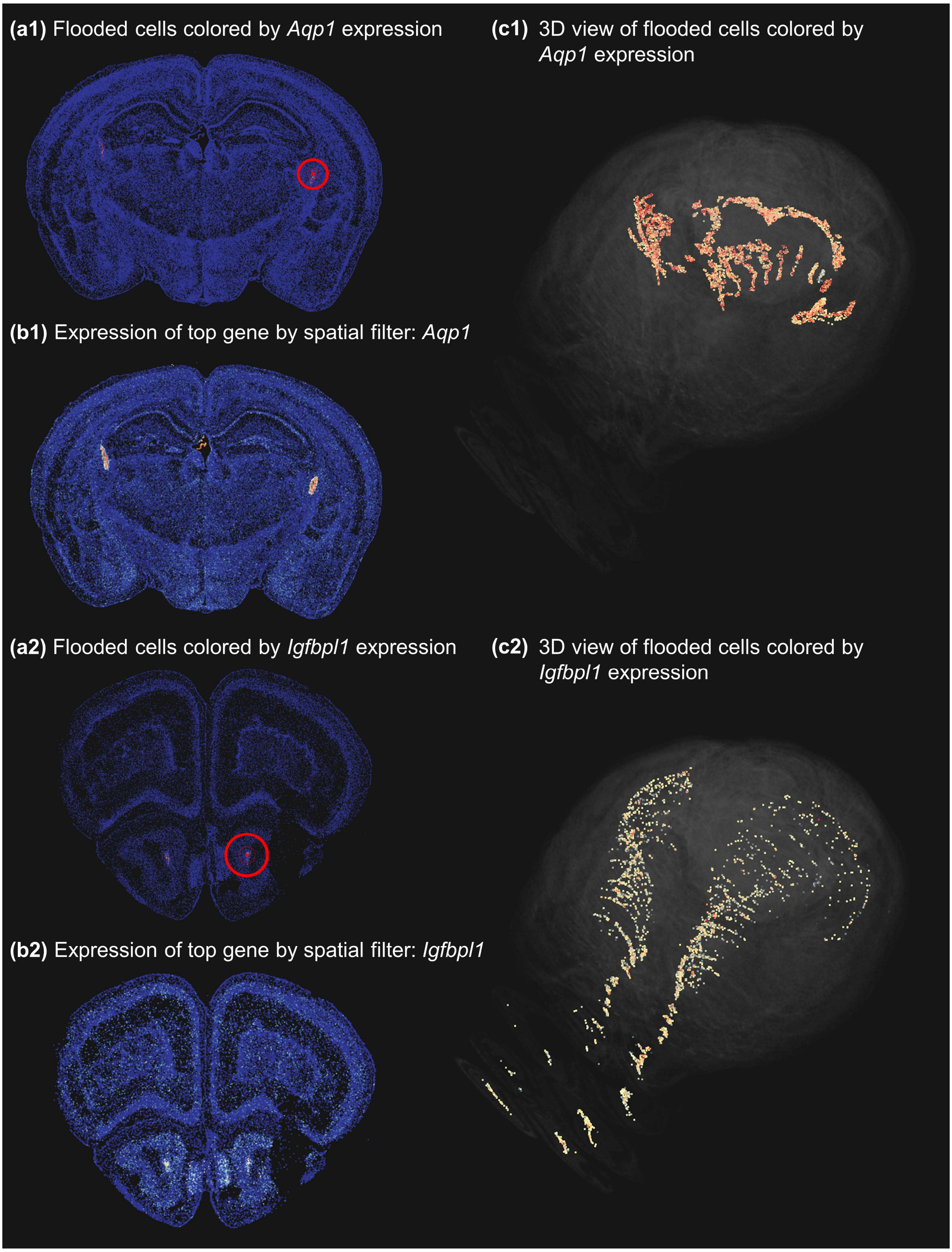

Supplementary Figure 4: Results of 3D whole-brain multi-slice exploration of the ABC Atlas dataset, related to Figure 7. (a) Local neighborhood reprojections, flooded cells are colored by the expression of the top gene identified by spatial filtering at the specified location. Selected seed cell is marked with a red dot in the center of a red circle representing the outer radius of the spatial filter. (b) Spatial maps of the brain slice, colored by the expression of the top gene, as a reference. (c) 3D views of flooded cells, colored by the expression of the top gene. (a1)(b1) are derived from

brain section 27, where *Aqp1* is ranked as the top gene at the cursor position. (a2)(b2) are derived from section 46, where *Igfbpl1* is ranked as the top gene at the cursor position. A red-blue color map is used to indicate gene expression levels, with red representing a high expression value and blue representing a low expression value.

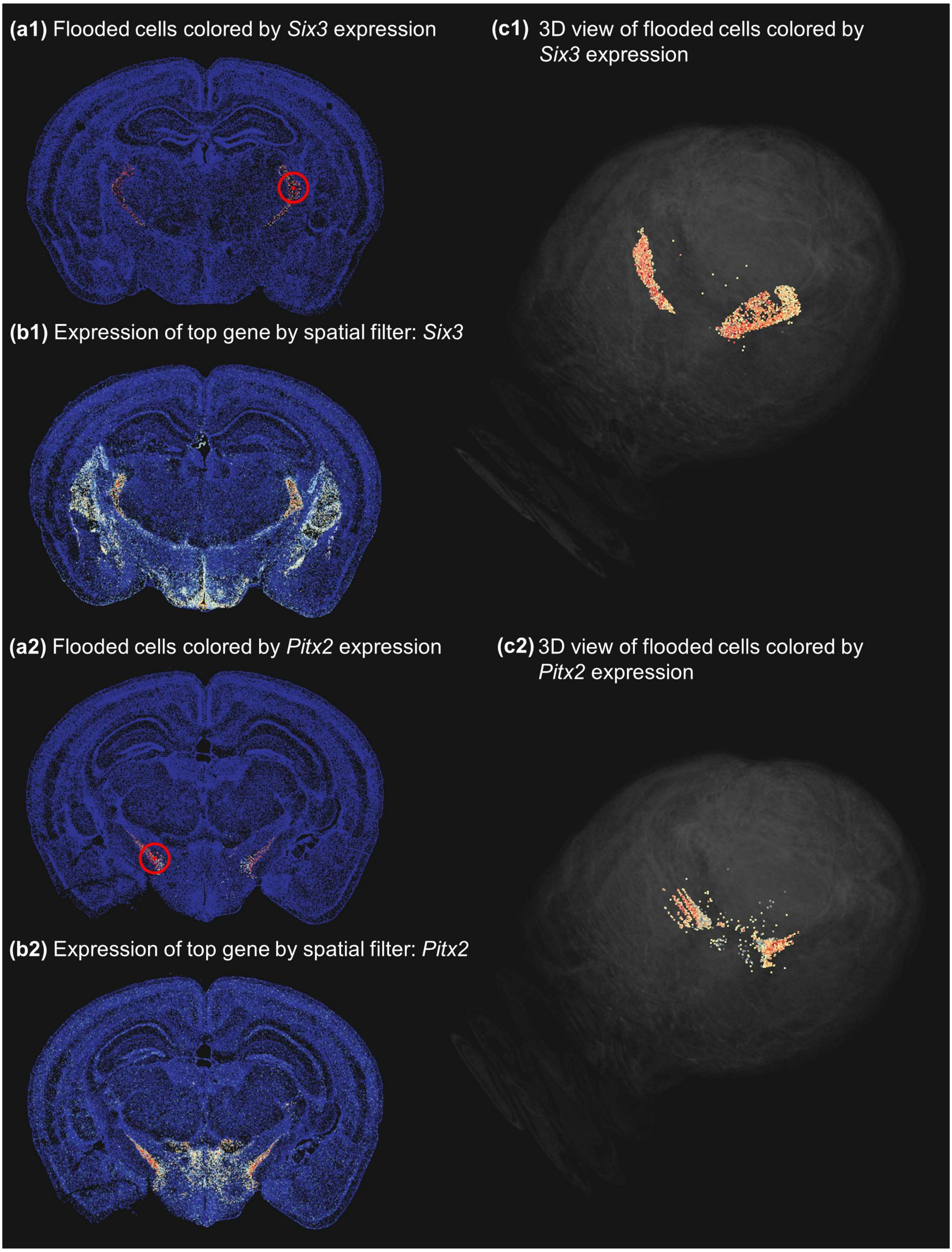

Supplementary Figure 5: Results of 3D whole-brain multi-slice exploration of the ABC Atlas dataset, related to Figure 7. (a) Local neighborhood reprojections, flooded cells are colored by the expression of the top gene identified by spatial filtering at the specified location. Selected seed cell is marked with a red dot in the center of a red circle representing the outer radius of the spatial filter. (b) Spatial maps of the brain slice, colored by the expression of the top gene, as a reference. (c) 3D views of flooded cells, colored by the expression of the top gene. (a1)(b1) are derived from

brain section 27, where *Six3* is ranked as the top gene at the cursor position. (a2)(b2) are derived from section 25, where *Pitx2* is ranked as the top gene at the cursor position. A red-blue color map is used to indicate gene expression levels, with red representing a high expression value and blue representing a low expression value.

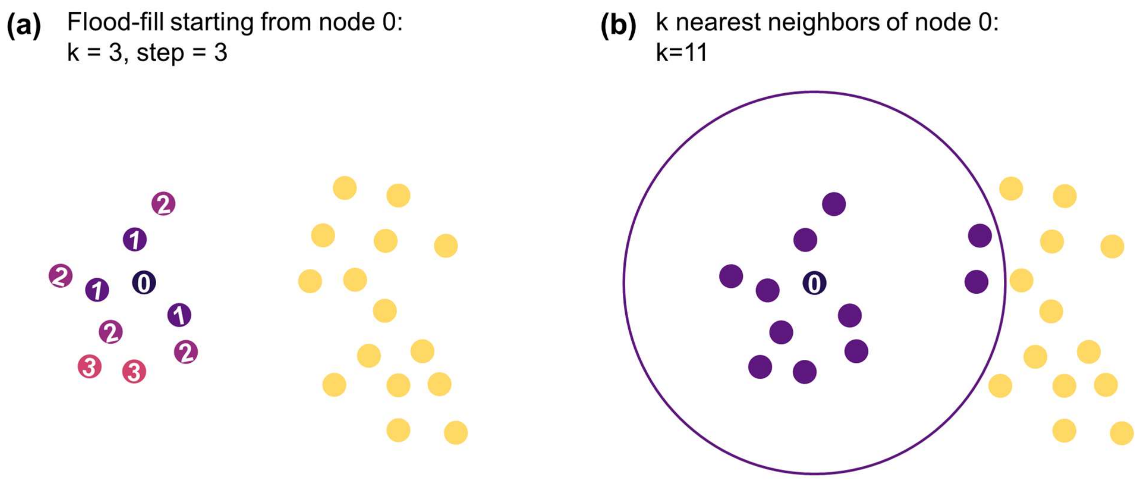

Supplementary Figure 6: Comparison between flood-filled neighbors (a) and direct nearest neighbors (b), related to STAR Methods. The selected neighbors are colored purple and the others are colored yellow. Numbers on the flood-filled nodes indicate the step index of flood-filling.

Supplementary Table 1: Performance of SpaceWalker on different datasets, related to STAR Methods. All experiments below were conducted on a 6-core, 2.60GHz Intel processor with 16GB of RAM and an NVIDIA GTX 1660Ti GPU (except the precomputation of neighborhood graph for the ABC Atlas).

	SpaceTx smFISH	SpaceTx MERFISH	HybISS	EEL FISH	ABC Atlas
Data dimension [cells,genes]	[2360, 314]	[2150, 314]	[4628, 117]	[127591, 440]	[3741416, 550]
Pre-processing	0.014 s	0.015 s	0.013 s	0.485 s	66.76 s
Precomputation of neighborhood graph	<1 s	<1 s	<1 s	51 s	15 min * (RTX 3070 Ti)
Memory usage	0.4 GB	0.4 GB	0.5 GB	1.5 GB	8.5 GB

*: The neighborhood graph for the ABC Atlas was precomputed on a RTX 3070 Ti GPU. Due to the large size of the ABC Atlas data, we suggest precomputing the neighborhood graph on the GPU. Once computed, it can be loaded into SpaceWalker. This approach ensures that the neighborhood graph only needs to be computed once. If not specified, the neighborhood graph was computed on the CPU.

Supplementary Table 2: Comparison between SpaceWalker and existing methods, related to STAR Methods.

	Clustering	Neighborhood	Gene identification	Interactivity
HMRF[7]	yes	spatial neighborhoods used for clustering	genes with expression patterns related to clusters	-
SC-MEB[8]	yes	spatial neighborhoods used for clustering	genes with expression patterns related to clusters	-
BayesSpace[9]	yes	spatial neighborhoods used for clustering	genes with expression patterns related to clusters	-
SpatialDE[10]	yes	-	genes with spatial expression patterns	-
Trendsceek[11]	-	-	genes with spatial expression patterns	-
Giotto[12]	yes	construction of nearest neighbor graph	genes with expression patterns related to clusters; SpatialDE, trendsceek etc.	interactive viewer
squidpy[13]	yes	construction of nearest neighbor graph	genes with spatial expression patterns	interactive viewer
CELLxGENE[17]	yes	-	genes between selected cell populations	interactive viewer
stLearn[24]	yes	construction of nearest neighbor graph	genes with spatial expression patterns	interactive viewer
SpaceWalker	-	construction of user-defined localized neighborhoods	genes with localized gene expression patterns at user-defined location, both spatially and high-dimensionally	interactive viewing in 2D and 3D, neighborhood searching and gene identification

Supplementary Table 3: Default parameters and the parameters that were used for all datasets in this work, related to STAR Methods.

		Default	SpaceTx smFISH and MERFISH	HybISS	EEL FISH	ABC Atlas
Neighborhood distance metrics (for local dimensionality and flood-fill)		Gene<200: manhattan distance; Gene>200: angular distance	Manhattan distance	Manhattan distance combined with a shared distance metric	Angular distance	Angular distance
Neighborhood size for intrinsic dimensionality		30	30	30	300/1000 (Figure 6)	30
Flood-fill	k (number of nodes in each flooding wave)	10	10	10	10	10
	Number of flooding steps	10	10	10	10 (different values used in videos)	10
Spatial filter	If restrict to flooded nodes	Yes	No (Figure 4, Supp. Figure 1) Yes (Supp. Figure 2-3)	No	No	Yes
	Inner radius	2.5	8	2	2	1.5
	Outer radius	5	16	5	5	4
HD filter	Inner radius	5	5	-	-	-

Note: the radii of the spatial filter used for the ABC Atlas is always the same in this paper, the outer radii of the spatial filter may look different in Figure 7 and Supplementary Figure 4-5 due to the different size of the brain slices.
